# Destabilization of Akt Promotes the Death of Myeloma Cell Lines

**DOI:** 10.1155/2014/190629

**Published:** 2014-08-31

**Authors:** Yanan Zhang, Yunfeng Fu, Fan Zhang, Jing Liu

**Affiliations:** Department of Hematology, The Third Xiangya Hospital, Central South University, Changsha, China

## Abstract

Constitutive activation of Akt is believed to be an oncogenic signal in multiple myeloma and is associated with poor patient prognosis and resistance to available treatment. The stability of Akt proteins is regulated by phosphorylating the highly conserved turn motif (TM) of these proteins and the chaperone protein HSP90. In this study we investigate the antitumor effects of inhibiting mTORC2 plus HSP90 in myeloma cell lines. We show that chronic exposure of cells to rapamycin can inhibit mTORC2 pathway, and AKT will be destabilized by administration of the HSP90 inhibitor 17-allylamino-geldanamycin (17-AAG). Finally, we show that the rapamycin synergizes with 17-AAG and inhibits myeloma cells growth and promotes cell death to a greater extent than either drug alone. Our studies provide a clinical rationale of use mTOR inhibitors and chaperone protein inhibitors in combination regimens for the treatment of human blood cancers.

## 1. Introduction

Constitutive activation of the AGC kinase PKB/Akt is believed to be an oncogenic signal in multiple myeloma and is associated with poor patient prognosis and resistance to available treatment [1, 2]. Constitutive phosphorylation of Akt leads to activation of downstream substrates involved in cell cycle regulation and apoptosis prevention [3]. It is already proved that Akt activation promotes tumor-cell proliferation by phosphorylating and inhibiting the cell-cycle inhibitor p27^Kip1^ and the F-box-containing transcription factor FoxO1 [4–6], as well as the proapoptotic protein BAD [7]. Akt activity also inhibits GSK3 resulting in suppressing the degradation of the antiapoptotic protein Mcl-1 [8, 9]. Extracellular stimulants can activate AKT through both growth factor dependent and growth factor independent ways by mammalian target of rapamycin complex 2 (mTORC2) [10–12].

Mammalian TORC2 is composed of mTOR, Rictor, mitogen-activated protein kinase associated protein 1 (Mapkap1/Sin1), mLST8, protein observed with Rictor (Protor/PRR5), and DEP domain containing mTOR interacting protein (DEPTOR) [13]. Pharmacologic or genetic inhibition of mTORC2 components impairs growth factor dependent Akt S473 phosphorylation and Akt signaling [10, 12, 14, 15]. Mammalian TORC2 also regulates the stability of Akt and cPKC proteins in a growth factor independent manner [16]. Mammalian TORC2 is required for the phosphorylation of Akt and cPKC at the turn motif (TM) site [12, 16]. Mammalian TORC2 interacts with actively translating ribosomes and phosphorylates the TM site of newly synthesized Akt and cPKC polypeptides during translation [17], which promotes the proper folding of newly synthesized Akt or cPKC polypeptides. However, the stability of Akt proteins may be rescued by association with the chaperone protein HSP90 when Akt is lacking TM phosphorylation [16]. Inhibition of HSP90 in *Sin*1^−/−^ mouse leukemia cells results in the reduction of Akt protein expression and promotes cell death [18].

Because of the crucial role of Akt in multiple myeloma biology, we decided to investigate the idea whether inhibition of both mTORC2 and HSP90 in multiple myeloma cells would decrease Akt expression and inhibit tumor cell proliferation and survival. We tested this novel therapeutic strategy by exploring the effect of rapamycin and 17-AAG in two different human multiple myeloma cell lines on the Akt expression, cell proliferation, and survival. We show that chronic rapamycin treatment inhibits mTORC2 on both cell lines, and coadministration of rapamycin and 17-AAG inhibits Akt expression and cell survival. These data reveal that combining the chaperone protein inhibitor with mTOR inhibitors can be considered as a promising new antineoplastic strategy.

## 2. Materials and Methods

### 2.1. Cell Lines and Culture

Multiple myeloma cell line KM3 was kindly provided by Professor Jian Hou from The Second Shanghai Military Medical University; multiple myeloma cell line U266 was kindly provided by Professor Jiankai Shen from The Second Xiangya Hospital. All cell lines were maintained in RPMI 1640 with 10% FBS, 100 U/mL penicillin, and 100 *μ*g/mL streptomycin at 37°C in 5% CO_2_ incubator. Cells were monitored daily and fresh medium was exchanged when needed. Cells were placed in a 6-wells plate; 17-AAG and rapamycin were added to the medium to obtain the desired final concentrations.

### 2.2. Reagents

#### 2.2.1. Antibodies

Anti-phospho-Akt T450 was purchased from Abcam Technology. Anti-phospho-Akt S473, anti-pan-Akt, anti-phospho-S6 S235/236, and anti-*β*-actin were purchased from Cell Signaling Technology. Reagents used for flow cytometry were BD Pharmingen FITC Annexin-V Apoptosis Detection Kit I purchased from BD company. All reagents were used at a 1 : 100 dilution of the stock from the same company.

#### 2.2.2. Inhibitors

Both 17-AAG and rapamycin were purchased from Gene Operation.

Stocks were prepared in DMSO at concentration of 40 *μ*mol/L, and 17-AAG was used at a final concentration of 600 nmmol/L; rapamycin was used at a final concentration of 20 nmmol/L. Drugs were diluted in culture medium with DMSO (<0.1%) immediately before use. Diluted drugs were used within 2 hours.

### 2.3. Determination of Cell Viability and Apoptosis

Cell viability was determined by the trypan blue dye exclusion. For a trypan blue staining, we diluted 1 part of the 0.4% prepared trypan blue to 9 parts of medium, which contained multiple myeloma cells, then mixed them and incubated them for 5 minutes. At last we counted cell percentage which was dyed into blue with optical microscope. Cells were counted in duplicate samples. To estimate apoptosis, control or treated cells were incubated with propidium iodide (PI) and Annexin V-FITC, following the protocol from the kit (FITC Annexin V Apoptosis Detection Kit I, BD Company), and then analyzed by flow cytometry. In brief, cells from 48-hour cultures were washed with ice-cold PBS and resuspended in binding buffer 100 *μ*L. Multiple myeloma cells were first incubated with 5 *μ*L Annexin V-FITC for 15 minutes at 4°C then incubated with 5 *μ*L PI just before analysis. All cells were washed and resuspended in FACS buffer for acquisition on FACSCalibur (BD Bioscience, CA) using CellQuest software (BD Bioscience, CA). Postacquisition analysis was performed with FlowJo software (Treestar, CA). Annexin V-positive and PI-negative cells reflect cells in the early stages of apoptosis, whereas Annexin V-positive and PI-positive cells reflect dead cells or cells at the late stages of apoptosis.

### 2.4. Immunoblotting

Cells were washed with PBS and lysed in RIPA buffer containing 50 mM Tris-HCI pH 8.0, 150 mM NaCl, 1% Triton X-100, 1% Na-deoxycholate, 0.1% SDS, 1 mM EDTA, 1 mM EGTA, 1 mM PMSF, 10 *μ*g/mL aprotinin, 10 *μ*g/mL leupeptin, 25 mM NaF, 1 mM Na_3_VO_4_, 25 mM *β*-glycerophosphate, and 2.5 mM p-nitrophenyl phosphate. Total cell lysates were resolved on 8% SDS-PAGE gels and transferred to an Immobilon-P membrane (Millipore, MA). The resulting blots were blocked with 5% nonfat dry milk and incubated with the antibodies overnight at 4°C as described previously. Antibody dilutions for blots ranged from 1 : 200 to 1 : 4000. Unbound primary antibody was removed by washing with TBS-T containing 0.1% Tween-20 and blots were incubated with anti-rabbit immunoglobulin conjugated with horseradish peroxidase and then developed using an enhanced chemiluminescence kit (Pierce ECL Plus Western Blotting Substrate, Thermo Scientific Pierce) following the manufacturer's instructions. The film was scanned and analyzed with Image-Pro Plus version 6.0 software. Blots were stripped and reprobed with anti-actin antibody (1 : 3000) to ensure equivalent protein loading. Different time points were chosen to determine the effect of the agents on phosphorylated proteins and total proteins (0–48 hours).

### 2.5. Statistical Analysis

Results are expressed as mean ± SD; the Student's *t*-test was used to determine the statistical significance of the differences between groups of samples. *P* < 0.05 was considered statistically significant. The number of sample replicates and the number of experimental replicates are indicated in the figure legends.

## 3. Results

### 3.1. Chronic Exposure to Rapamycin Inhibits mTORC2 Pathway on U266 and KM3 Cell Lines

**Figure 1:**
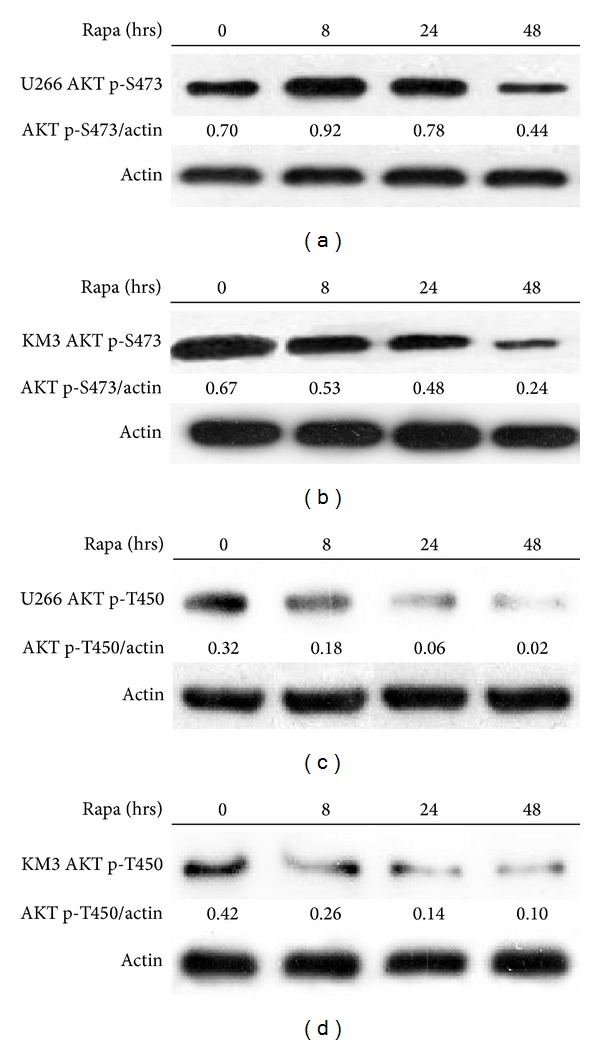
Prolonged exposure to rapamycin also inhibits mTORC2 pathway. (a) U266 cells or (b) Km3 cells were cultured in the presence of 20 nmmol/L rapamycin for the indicated periods of time. Total cellular proteins were assayed by immunoblotting for Akt p-S473 phosphorylation. Actin expression serves as a loading control. The Akt p-S473/actin ratio was calculated by dividing the total pixel volume of Akt by the total pixel volume of actin. (c) U266 cells or (d) Km3 cells were cultured in the presence of 20 nmmol/L rapamycin for the indicated periods of time. Total cellular proteins were assayed by immunoblotting for Akt p-T450 phosphorylation. Actin expression served as a loading control. The Akt p-T450/actin ratio was calculated by dividing the total pixel volume of Akt by the total pixel volume of actin. The results shown are representative of three independent experiments.

mTOR1 regulates various aspects of protein synthesis, which connects mTOR1 to many physiological processes such as nutrient, stress, and hormone signaling [19–22]. In blood cancers mTOR signaling pathway is commonly activated to promote uncontrolled cellular growth and proliferation. During cellular protein translational controls, mTOR1 is one of the rate-limiting signal nodes. And moreover mTORC2 plays an important role in the dynamic interaction between tumor cells and BM microenvironment [23, 24], which is crucial in myeloma pathogenesis and resistance to treatment. Rapamycin is one of the most classical mTORC1 inhibitors. Usually people think that rapamycin (and its analogs) cannot completely inhibit TOR2 pathway in most cells. Recently, it has been proved that chronic exposure of certain kinds of cells to rapamycin can inhibit mTORC2 pathway, but the precise mechanism is still unclear [25, 26]. To address whether chronic exposure to rapamycin can inhibit mTORC2 pathway on myeloma cell, we cultured our two myeloma cell lines (U266 and KM3) in the presence of 20 nM rapamycin up to 48 h and harvested the cell lysate at 0 h, 8 h, 24 h, and 48 h. We found that after 48 h treatment rapamycin was able to inhibit Akt S473 phosphorylation, in both cell lines (Figures 1(a) and 1(b)), and Akt T450 phosphorylation (Figures 1(c) and 1(d)), which is the well-known downstream of mTORC2. We also found that rapamycin inhibits mTORC1-dependent S6 S235/236 phosphorylation at 48 h (data not shown) as previously described.

### 3.2. Combined 17-AAG and Prolonged Rapamycin Treatment on Myeloma Cell Lines Destabilizes Akt

**Figure 2:**
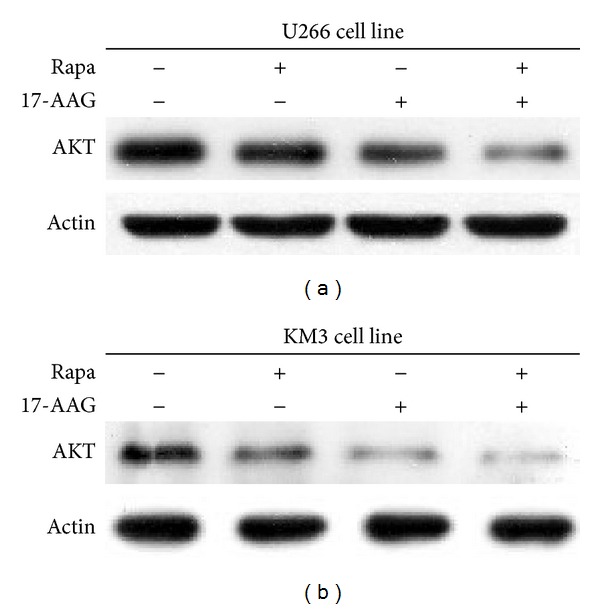
Coadministration of Rapa and 17-AAG destabilizes Akt. (a) U266 cells and (b) KM3 cells were treated with vehicle (Ctrl), 20 nmmol/L rapamycin, 600 nmol/L 17-AAG, or rapamycin plus 17-AAG for 48 h. Total Akt expression was measured by immunoblotting. Actin expression served as a loading control. The results shown are representative of three independent experiments.

Heat shock protein 90 can protect newly synthesized folded kinases when cells lack TM phosphorylation [27]. As previous results already show that the stability of Akt in mice MEF cells [16] and mice leukemia cells [18] is maintained by the TM phosphorylation and HSP90, we ask what would happen to Akt levels of myeloma cells if we inhibit both mTOR2 and Hsp90. We treated U266 and KM3 cells with combined 17-AAG and prolonged rapamycin for 48 h, which led to a significant decrease in Akt protein level (Figures 2(a) and 2(b)).

### 3.3. Coadministration of Rapamycin and 17-AAG Promotes Death of Myeloma Cell Lines

**Figure 3:**
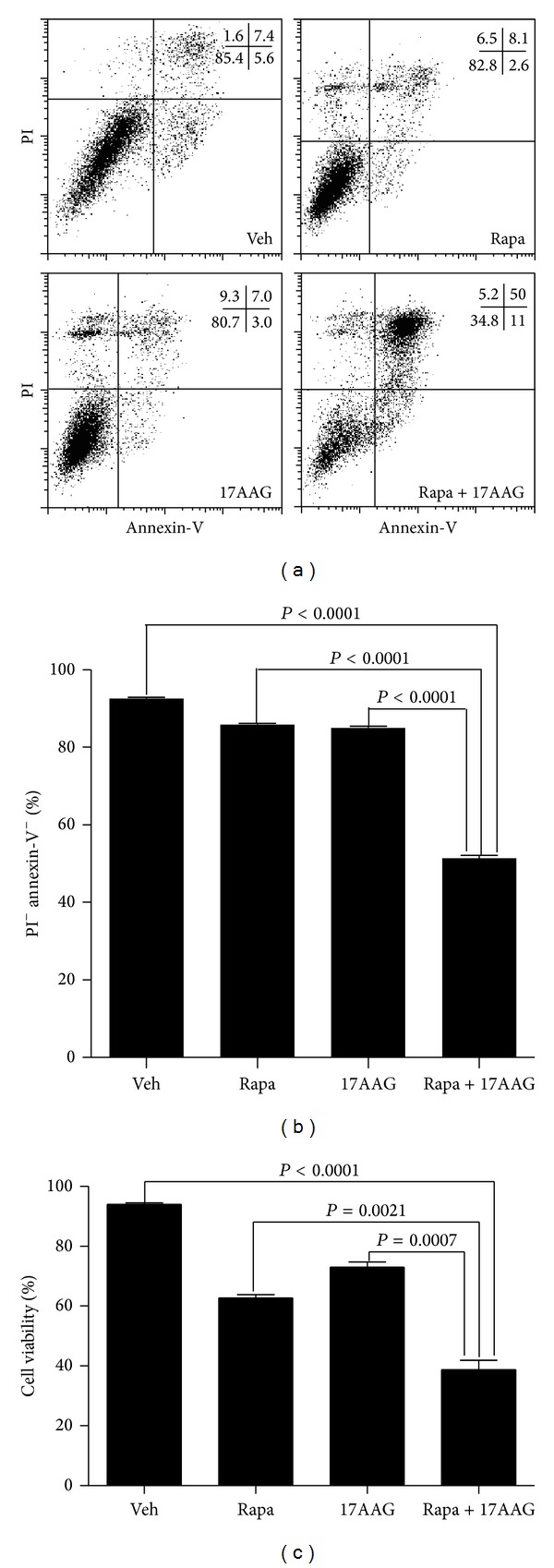
Coadministration of Rapamycin and 17-AAG promotes km3 cell line death. (a) KM3 cells were cultured for 48 h with vehicle (Ctrl), 20 nmmol/L rapamycin, 600 nmol/L 17-AAG, or rapamycin plus 17-AAG, and cells viability was measured by flow cytometry with PI and Annexin-V staining. A representative FACS plot is shown. The numbers in the plot show percentages of the gated populations in each quadrant. (b) It is the average of triplicate samples of data (a) from 1 of 3 independent experiments. (c) KM3 cells were cultured for 48 h with vehicle (Ctrl), 20 nmmol/L rapamycin, 600 nmol/L 17-AAG, or rapamycin plus 17-AAG, and viable cells were determined by trypan blue exclusion assay. The data shown are the average of triplicate samples from 1 of 3 independent experiments. The *P* values shown were calculated by a two-tailed test.

Since Akt is critical for the survival of tumor cells, we decided to explore if chronic exposure of cells to rapamycin, the pharmacologic method of mTORC2 inhibition, could synergize with 17-AAG to suppress myeloma cell lines proliferation in vitro. We first compared the cell viability of KM3 cells treated with rapamycin alone, 17-AAG alone, or rapamycin plus 17-AAG with untreated cells. Cell viability was assessed by PI and Annexin-V staining and flow cytometry. We observed that the KM3 cells resulted in a 50% increase in proportion of viable cells after rapamycin and 17-AAG treatment for 48 hrs in comparison with the cells treated with single drugs (Figures 3(a) and 3(b)). We also determined the live cells percentage by trypan blue exclusion, which gives us the same results as FACS (Figure 3(c)).

**Figure 4:**
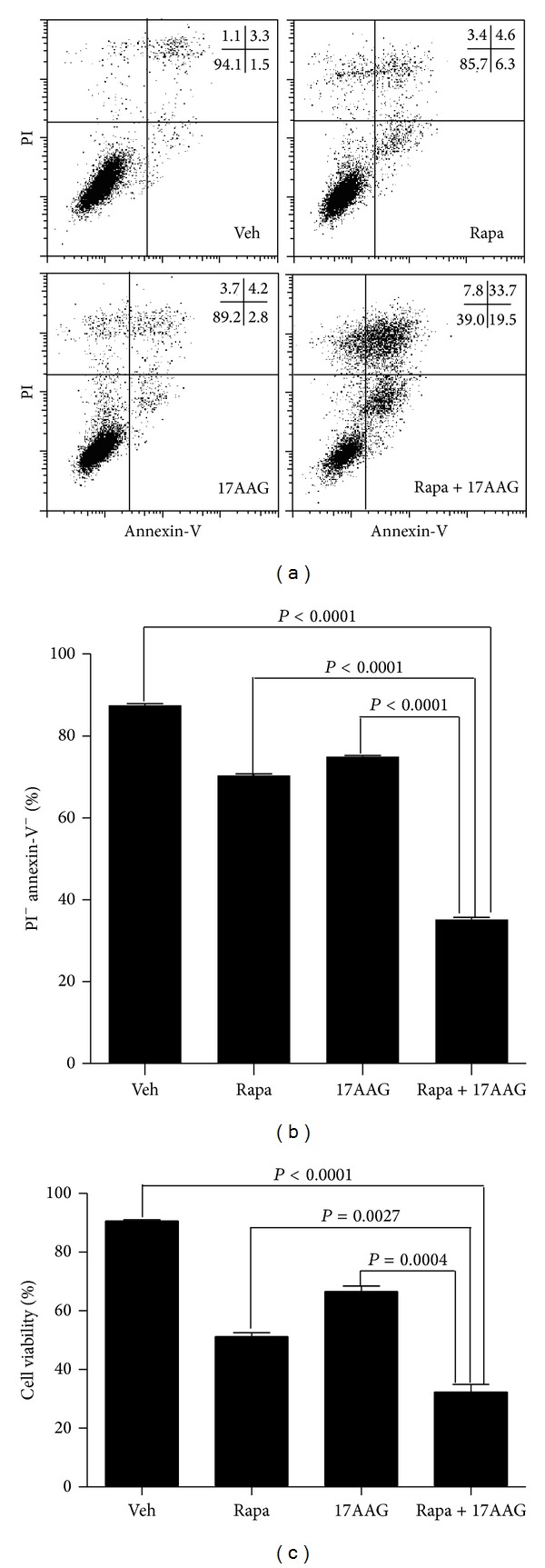
Coadministration of Rapa and 17-AAG promotes U266 cell line death. (a) U266 cells were cultured for 48 h with vehicle (Ctrl), 20 nmmol/L rapamycin, 600 nmol/L 17-AAG, or rapamycin plus 17-AAG, and cells viability was measured by flow cytometry with PI and Annexin-V staining. A representative FACS plot is shown. The numbers in the plot show percentages of the gated populations in each quadrant. (b) It is the average of triplicate samples of data (a) from 1 of 3 independent experiments. (c) U266 cells were cultured for 48 h with vehicle (Ctrl), 20 nmmol/L rapamycin, 600 nmol/L 17-AAG, or rapamycin plus 17-AAG, and viable cells were determined by trypan blue exclusion assay. The data shown are the average of triplicate samples from 1 of 3 independent experiments. The* P* values shown were calculated by a two-tailed test.

Consistently, cotreatment of U266 cells with rapamycin plus 17-AAG resulted in a significantly greater inhibition of myeloma cell growth when compared to each drug alone (Figures 4(a), 4(b), and 4(c)). These data show that 17-AAG and prolonged rapamycin treatment act in synergy to inhibit myeloma cell proliferation and survival.

## 4. Discussion

The AGC kinase PKB/Akt is constitutively activated in human myeloma cell lines and freshly isolated plasmocytes from patients with MM [28] and is considered as an oncogenic signal in MM. It is associated with poor patient prognosis and resistance to available treatment [1, 2]. Therefore, it is a logical strategy to include the inhibition of Akt activity in the treatment of MM. Full Akt activation requires phosphorylation at residues of both S473 and T308. mTOR1 regulates the activation of Akt through the phosphorylation of residue T308, but prior studies using TORC1 inhibitors have shown limited effect in complete inhibition of Akt. mTORC2 can phosphorylate Akt HM site at Ser473 through the growth factor dependent pathway, but there are also studies showing that the inhibition of Akt HM phosphorylation does not fully suppress Akt signaling [10, 12, 25, 29]. However, mTORC2 can phosphorylate the TM of Akt (T450) and cPKC proteins [16, 30], which is essential to maintain the stability of Akt and cPKC. In the absence of mTORC2, Akt, and cPKC TM phosphorylation is abolished and the stability of these proteins is reduced [16, 30]. Destabilization of Akt proteins gives us a totally new approach to induce an anticancer effect.

Rapamycin is one of the most classical mTORC1 inhibitors, which has been utilized therapeutically for years as immunosuppressant. Usually people think rapamycin (and its analogs) cannot completely inhibit TOR2 pathway in most cells. Recently, it has been proved that chronic exposure of certain kinds of cells to rapamycin can inhibit mTORC2 pathway, though the precise mechanism is unclear [12, 26]. In our study, after myeloma cell lines were treated in the presence of rapamycin extended to 48 h, two important mTORC2 downstream targets—Akt S473 and T450 phosphorylation—were inhibited, which made us believe that myeloma cells mTORC2 pathway was inhibited after chronic exposure to rapamycin.

When Akt lack TM phosphorylation, HSP90 associates with Akt proteins and rescues the stability of the newly synthesized Akt proteins [16]. In this study, we utilized the ability of chronic rapamycin treatment to inhibit mTORC2 in myeloma cells. We show that the treatment of* myeloma* cells with prolonged rapamycin and HSP90 inhibitor 17-AAG results in the rapid loss of total Akt protein expressed in both myeloma cell lines (Figure 2). Overall, our data demonstrate that the combined inhibition of mTORC2 and HSP90 promotes the destabilization of Akt proteins and increases the capacity of mTOR inhibitors and 17-AAG to elicit an antimyeloma effect.

Due to the critical role of Akt in regulating cell survival, we predicted that prolonged rapamycin and 17-AAG dependent reduction of Akt expression would promote the cell death of* myeloma* cells. Indeed, prolonged rapamycin and 17-AAG treatment induced substantially more cell death than any single drug treatment. These data provide strong in vitro evidence and suggest that the dual inhibition of mTOR plus HSP90 may serve as an effective antimyeloma therapy.

Recently, one of our authors reported that the HSP90 inhibitor 17-AAG induced tumor regression when combined with pharmacologic and genetic inhibition of mTORC2 in a mouse model of leukemia cells [18]. In that study the author first presented in vitro data showing that 17-AAG treatment induced substantial Akt expression reduction and more cell death in *Sin*1^−/−^ pre-B leukemia cells than wild type pre-B leukemia cells. After reconstitution of human Sin1 in *Sin*1^−/−^ pre-B leukemia cells, there was increased resistance to 17-AAG mediated cell death. Then in vivo experiments supported these in vitro studies. Authors transplanted *Sin*1^+/+^ or *Sin*1^−/−^ p210 BCR-Abl transformed mouse leukemia cells into wild type mice and treated the recipients with 17-AAG or vehicle for five days. There were equivalent numbers of *Sin*1^+/+^ and *Sin*1^−/−^ leukemia cells recovered from the bone marrow and spleens of vehicle treated mice indicating Sin1 gene status does not alter leukemia cells growth. However, after 17AGG treatment, *Sin*1^−/−^ pre-B cell tumor burden was significantly reduced both in the bone marrow and in the spleen while the *Sin*1^+/+^ leukemia cell numbers were not varied much by 17-AAG. Authors also presented data showing that 17-AAG synergized with rapamycin induced a cytotoxic response causing leukemia cells regression. These data provide strong evidence that the inhibition of mTORC2 sensitizes leukemia cells to 17-AAG.

Our data and leukemia mouse model indicate that inhibition of both mTORC2 and HSP90 will produce a synergistic antitumor effect which is more superior to the inhibition of the mTOR or chaperon pathway alone. The directly targeted mTORC2 inhibitors are currently under development but have not yet been approved for clinical use. Rapamycin (and its analogues) cannot directly inhibit mTORC2 pathway, but our research indicates that chronic rapamycin treatment may block mTORC2 complex assembly in myeloma cell lines U266 and KM3 as in many other cell types [12, 16, 26, 31]. Therefore it is hopeful to propose an antimyeloma strategy by rapamycin treatment synergized with HSP90 inhibitors such as 17-AAG. Furthermore, considering the immunosuppressive and metabolic side effects of rapamycin, we predict that molecules directly inhibiting mTORC2 will be an important new target which can be used in combination with chaperon inhibitors to achieve better cure outcomes for patients with hematologic malignancies.
